# Bioprospecting of microbial strains for biofuel production: metabolic engineering, applications, and challenges

**DOI:** 10.1186/s13068-020-01853-2

**Published:** 2021-01-06

**Authors:** Mobolaji Felicia Adegboye, Omena Bernard Ojuederie, Paola M. Talia, Olubukola Oluranti Babalola

**Affiliations:** 1 Food Security and Safety Niche Area, Faculty of Natural and Agricultural Sciences, North-West University, Mmabatho, Private Bag X2046, 2735 South Africa; 2 Department of Biological Sciences, Faculty of Science, Kings University, Ode-Omu, PMB 555, Osun State Nigeria; 3 Instituto de Agrobiotecnología y Biología Molecular (IABIMO), Instituto Nacional de Tecnología Agropecuaria (INTA CICVyA, CNIA, INTA Castelar, Dr. N. Repetto y Los Reseros s/n, (1686) Hurlingham, 1686) Hurlingham, Provincia de Buenos Aires, Argentina; 4 Consejo Nacional de Investigaciones Científicas Y Tecnológicas (CONICET), Buenos Aires, Provincia de Buenos Aires Argentina

**Keywords:** CRISPER/Cas9, Lignocellulose, Fermentation, Metabolic pathways, Microbial cell factories, Model strains

## Abstract

The issues of global warming, coupled with fossil fuel depletion, have undoubtedly led to renewed interest in other sources of commercial fuels. The search for renewable fuels has motivated research into the biological degradation of lignocellulosic biomass feedstock to produce biofuels such as bioethanol, biodiesel, and biohydrogen. The model strain for biofuel production needs the capability to utilize a high amount of substrate, transportation of sugar through fast and deregulated pathways, ability to tolerate inhibitory compounds and end products, and increased metabolic fluxes to produce an improved fermentation product. Engineering microbes might be a great approach to produce biofuel from lignocellulosic biomass by exploiting metabolic pathways economically. Metabolic engineering is an advanced technology for the construction of highly effective microbial cell factories and a key component for the next-generation bioeconomy. It has been extensively used to redirect the biosynthetic pathway to produce desired products in several native or engineered hosts. A wide range of novel compounds has been manufactured through engineering metabolic pathways or endogenous metabolism optimizations by metabolic engineers. This review is focused on the potential utilization of engineered strains to produce biofuel and gives prospects for improvement in metabolic engineering for new strain development using advanced technologies.

## Background

The continuous increase in global consumption of energy presently anticipated a rise in energy demand that will not be met in the short term. The depletion of fossil fuel reservoirs and climate change issues are strong indicators of the need for renewable and sustainable fuel alternatives [1]. Production of renewable fuels, biodegradable and environmentally friendly, is seen as a significant potential substitute for fossil fuel [2]. Lignocellulosic biomass serves as a reliable feedstock for renewable energy since it is admittedly not in competition with food. Lignocellulose biomass is cultivated primarily for biofuel production such as poplar, sunflower, and jatropha, which are used as feedstocks for biofuel production. They are found abundantly in nature and are available globally, making them an attractive source of biomass for biofuel production. They also have significant advantages over first-generation biomass feedstocks since they are not used as food sources [3, 4]. Biofuel produced from lignocellulosic feedstock has been proven to be environmentally friendly, helps reduce dependence on fossil fuel [5], serves as an alternative for declining petroleum reservoirs, and also provides an economic improvement, especially to rural communities [6].

Microbes from various habitats naturally produce a broad array of bioactive compounds that are used as fuels, drugs, and other important chemicals [7–9]. They have excelled at producing biofuel through the biosynthesis of enzymes that act on diverse feedstocks for many years under different processes [10]. Most strategies for converting lignocellulosic biomass to biofuels require the depolymerization of polysaccharides catalyzed by the action of specific enzymes. However, one of the key impediments for the development of an economically feasible lignocellulose-based biofuel industry is the cost of enzymes [11, 12]. Nonetheless, intensive studies are ongoing globally, towards increasing biofuel production whilst reducing the cost of production for sustainable industries [13–16]. Microbial strain development and improvement through genetic engineering and optimization of fermentation parameters have augmented the production of biofuel. Although one of the main drawbacks is the method for optimizing various processes for maximal yield, the incorporation of process engineering, fermentation technology, enzyme engineering, and metabolic engineering has helped the industry tremendously.

The advent of metabolic engineering and the increase in the number of whole-genome sequenced organisms has contributed to improvement in the manipulation of microbial metabolic pathways and the production of numerous essential chemicals for the production of biofuel [17, 18]. The manipulation and evolution of different pathway enzymes also serve as a platform to increase the number and types of bioactive compounds that can be biosynthesized by microorganisms [19]. The biosynthesis of advanced biofuels such as alkanes, alkenes, and aromatics by microbes will involve an extensive manipulation of their metabolism. This review discusses the importance of model strains by metabolic engineering as a powerful tool to enhance biofuel production from lignocellulosic biomass and the challenges encompassed therein.

### Structure of lignocellulosic biomass feedstock

**Fig. 1:**
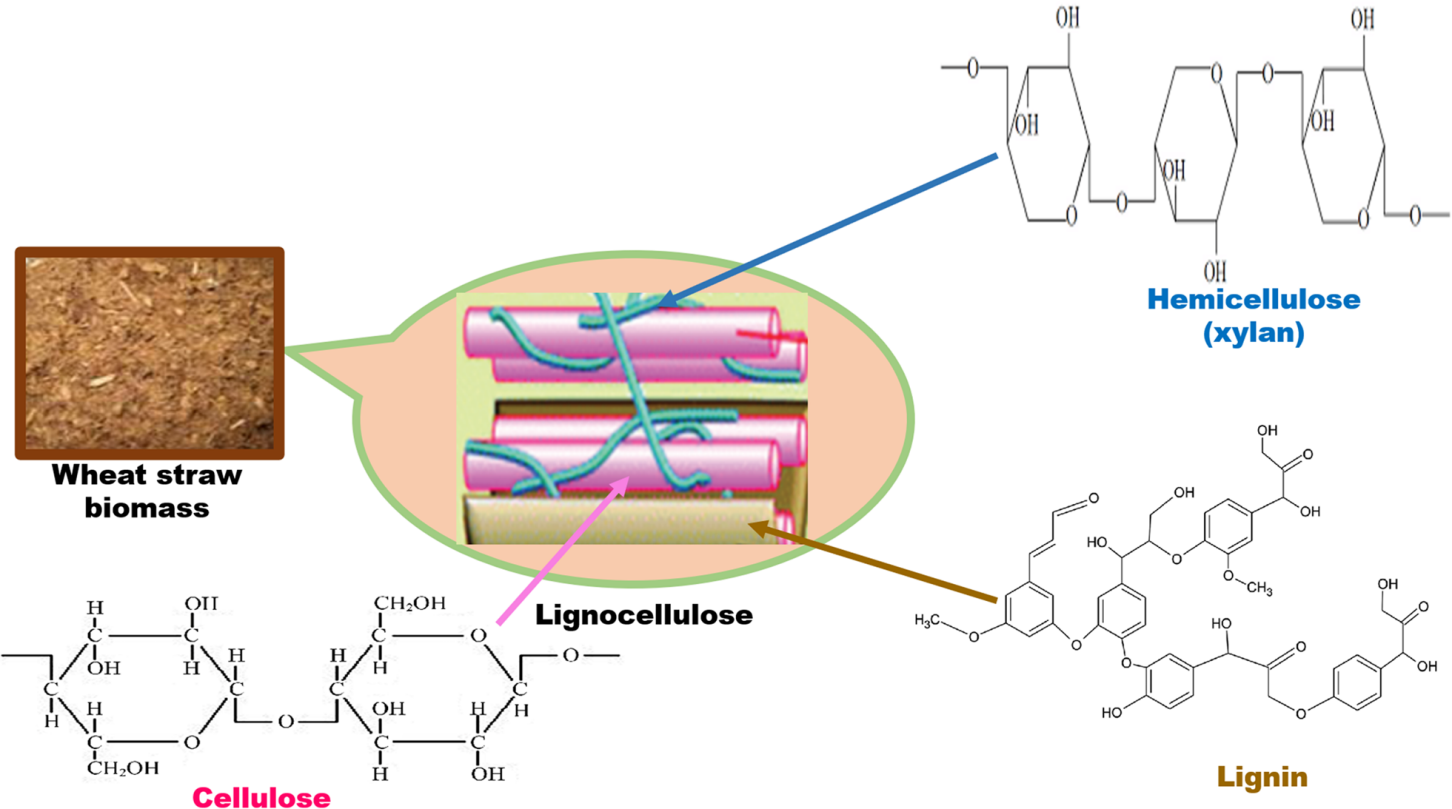
Lignocellulosic biomass structure

Lignocellulose constitutes the world’s largest biofuel renewable resource. They are the major source of underutilized feedstock, and their abundance negatively affects land use. Biomass feedstock from plants is naturally recalcitrant because of the complex polymer composition [20]. Lignocellulose, a complex carbohydrate polymer on a dry matter basis, comprises about 40–50% cellulose [(C_6_H_10_O_5_)n], 20–40% hemicellulose [(C_5_H_8_O_4_)m], 18–25% lignin [(C_9_H_10_O_3_(OCH_3_)0.9–1.7)x] and other extractable components [21]. The relative abundances of these three fractions are significant factors to be considered for probable energy production. Each component has a definite function in lignocellulose. Strength and flexibility are provided by cellulose, while hemicellulose acts as a link between lignin and cellulose fibers (Fig. 1). Apart from keeping cellulose and hemicellulose fibers glued together, lignin also gives structural support.

Cellulose is an unbranched crystalline biopolymer made up of several repeating glucose units linked by β-1,4 glycosidic bonds [22]. Cellulose has a molecular weight of 1621,406 g/mol. It is biosynthesized and found in the cell wall of plants joined by hydrogen bonding and van der Waals forces. Cellulose is insoluble in most solvents due to the strong hydrogen bonds and its fibrous nature [23]. Cellulose occurs in both crystalline and amorphous forms. In its crystalline form, the fibers are packed very tightly and practically inaccessible to enzymatic degradation.

Hemicellulose is a heteropolymer of several kinds of sugars (xylose, arabinose, rhamnose, galactose, and mannose). It may contain uronic acids, which are sugar acids known as d-glucuronic, d-galacturonic, and methylgalacturonic acids [24]. It is a short, amorphous, and highly branched polymer and its backbone chain comprises mainly xylan β (1 → 4) linkages [25]. Xylan is the predominant component in hemicellulose, but its composition varies from one feedstock to another. The molecular weight of hemicellulosic biomass is about 30,000 g/mol or less.

Lignin is a three-dimensional aromatic polymer of *p-*hydroxyphenylpropanoid units coupled together by C–C and C–O–C links [25, 26]. It is hydrophobic and is firmly bound to the two other carbohydrate polymers. Lignin is made up of three phenolic monomers of phenyl propionic alcohol namely, p-coumaryl, coniferyl, and sinapyl [24]. It contains methoxyl, phenolic, hydroxyl, and terminal aldehyde groups in the side chain and partially soluble in most organic solvents. The average molecular weight of lignin is about 20,000 [23]. Because of the diversity of the lignocellulose component and their recalcitrance, its complete hydrolysis into monomers is catalyzed by several enzymes. The complete utilization of these components would play an important part in the economic effectiveness of the lignocellulose in biofuel processes.

### Lignocelluloytic enzymes involved in polysaccharide biomass hydrolysis

Lignocellulosic biomass is the predominant and cost-effective renewable natural resource globally employed for biofuel production as a result of its high cellulose content [27]. Nonetheless, due to the recalcitrance nature of lignocellulose, its depolymerization is hindered. Lignocellulases such as cellulases, hemicellulases, pectinases, as well as lignases and polysaccharide oxygenases, are required to completely breakdown lignocellulose. These hydrolytic enzymes stimulate plant cell wall extension indirectly by decreasing the size and viscosity of matrix polymers, potentially augmenting the action of wall loosening agents [28, 29]. The cell wall of plants comprises cellulose and hemicellulose, which, when hydrolyzed, gives rise to fermentable sugars such as glucose, galactose, etc. which serves as a carbon source for the proliferation of microbes involved in biofuel production. Based on their structure and function, cellulases can be categorized into three types; (i) endoglucanases, (ii) exoglucanases, also known as cellobiohydrolases, and (iii) β-glucosidases, also called cellobiases [27]. These enzymes work in unison to hydrolyze cellulose in the cell wall of plants. Endoglucanases act by randomly attacking the internal sites of the amorphous part of cellulose, thereby paving the way for cellobiohydrolase action on the crystalline region of cellulose hydrolyzing it to cellobiose [30–32]. The synergistic action of endoglucanase and cellobiohydrolase produces cellobiose, which is then cleaved by β-glucosidases to glucose molecules. Microbes then utilize the energy stored in glucose converting it to hydrocarbon fuel through transforming the sunlight energy to chemical energy [33, 34]. The activities of the different cellulases are governed by their functional properties, which have been extensively reviewed by Obeng et al. [27]. Table 1 describes the various functional properties of the three groups of cellulases.

**Table 1:** Functional and structural properties of cellulases

Type of cellulase	EC Number	Functions	Structural properties	References
Endoglucanases	EC 3.2.1.4	Breaks internal linkages of cellulose molecules, producing cellobiose and possesses rapid dissociation capacity	They possess short loops that stick along cellulose chains to yield long-chain oligomers	[27, 39]
Exoglucanases or Cellobiohydrolases,	EC 3.2.1.74	Cleave the same glycosidic bond from terminal ends of cellulose molecules, producing cellobiose	They possess long loops and attracted to crystalline sites along cellulose microfibril chains and produce mainly Cellodextrin It exists in two forms based on the part of the oligosaccharide chain that is attacked. The reducing end and non-reducing end of cellobiohydrolase	[27, 39, 40]
β-glucosidases or cellobiases	EC 3.2.1.21	Cleaves cellobiose into two glucose molecules	It has a solid structure with the functioning site within a pocket which permits the entry of disaccharides. It has 2 forms which catalyze hydrolysis either from the reducing chain ends or the non-reducing chain ends	[22, 27, 41]

**Fig. 2:**
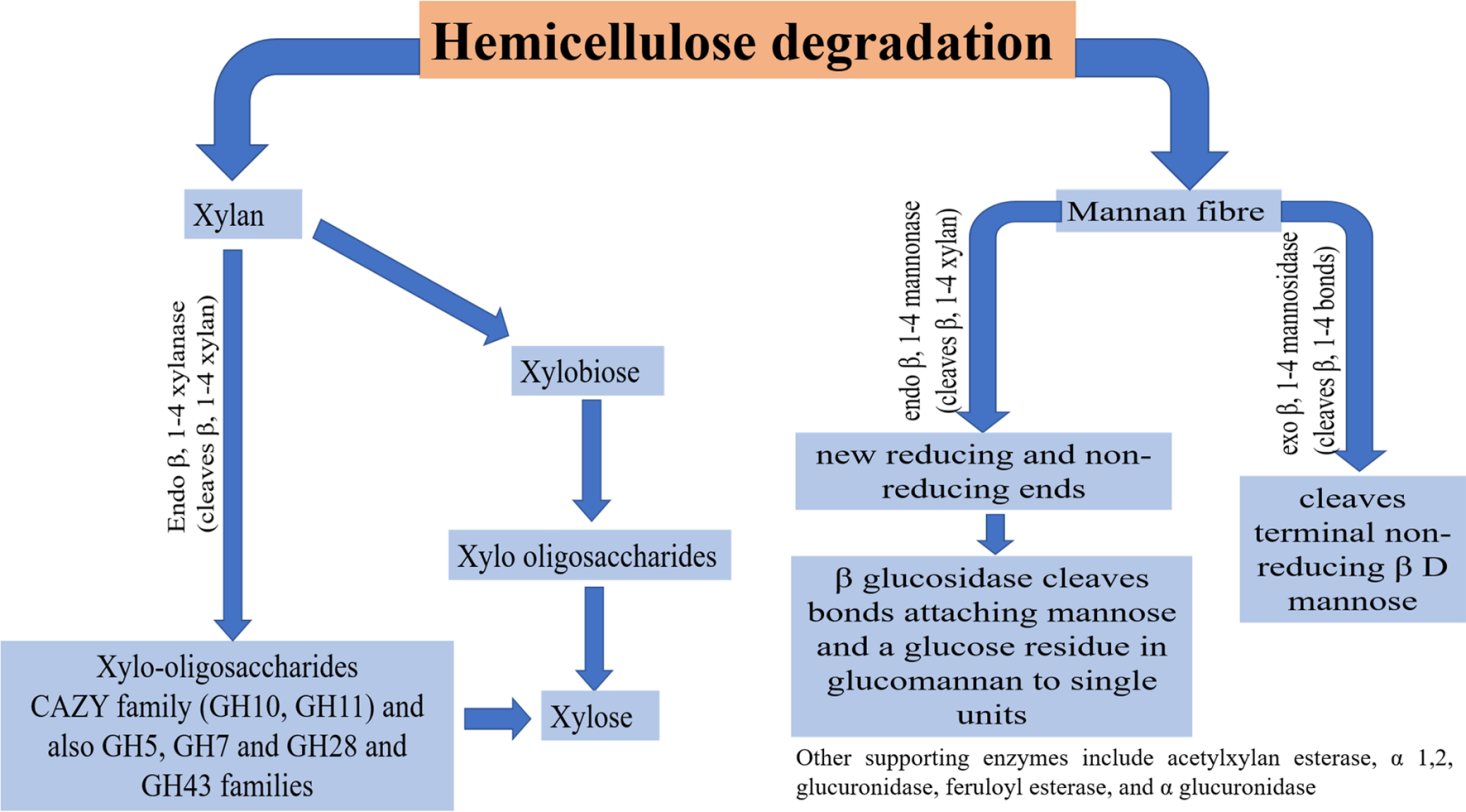
Degradation of hemicellulose by xylanases

Since hemicellulose is a heteropolymer consisting of a complex polysaccharide matrix composed of monomeric sugars and sugar acids linked together by β 1,4- and β1,3-glycosidic bonds, a combination of exo and endo-enzymes are required to completely cleave the internal bonds and set the monomeric sugars free [35, 36]. Hemicellulose is degraded by enzymes that act on xylan, degrading it to lower molecular weight oligosaccharides. The first of these enzymes is endo β-1,4 xylanase (E.C.3.2.1.8), which cleaves β-1,4 xylosidic bonds in xylan to xylo-oligosaccharides which is then converted to xylose, The other enzyme xylan β-1,4 xylosidase otherwise known as xylan β-1,4 xylosidase (E.C.3.2.1.37), cleaves xylobiose and smaller xylo-oligosaccharides to xylose (Fig. 2). These hemicellulose degrading enzymes are produced by bacterial and fungal species. For example, endo β-1,4 xylanase of the CAZy family (GH5, GH7, GH8, GH10, GH11, and GH43) are synthesized by fungi such as the *Trichoderma longibrachiatum*, *Aspergillus niger*, and *Ustilago maydis* [36–38].

Most of the xylanases fall under the GH10 and GH11 families, which differ in their substrate specificity, with GH10 having a wider specificity than GH11 endoxylanase family [42–44]. Other hemicellulolytic enzymes that work in synergism with the xylanase include β-mannanases and arabinofuranosidases, which play key roles in the cleavage of hemicelluloses composed of mannan or arabinofuranosyl facilitating the catalytic action of xylanase on xylan [45]. Bhardwaj et al. [46] recently gave an extensive review of microbial xylanases, highlighting the different families as well as its synthesis and applications in an emerging bioeconomy.

Apart from cellulose and hemicellulose, lignocellulosic biomass also contains little proportion of polysaccharides called pectin, which accounts for about 5% of total dry weight and is often found as a major component of agricultural wastes [47]. Pectin is composed of α-1,4-d-galacturonic acid linkages. These linkages are hydrolyzed by three different types of pectinases: hydrolases, lyases, and esterase based on their mode of action [48]. Hydrolases come in two forms depending on if the cleavage occurs within the molecule or at the terminal end.

Endopolygalacturonase (EC 3.2.1.15) hydrolyzes homogalacturonan in pectic acid and oligomers by releasing digalacturonic and galacturonic acid units from their reducing ends, while exopolygalacturonase (EC 3.2.1.67) acts on the reducing end of galacturonyl-oligomers produced by endopolygalacturonase, cleaving the α1,4-glycosidic bonds and subsequently releasing galacturonic acid from the non-reducing end [47, 49]. Esterase (EC 3.1.1.11) on the other hand, catalyzes the degradation of the methyl ester bonds in pectin by a de-esterification process, resulting in the production of pectic acid [48]. Hence, it is also called pectin methylesterase [50]. Lastly, lyase catalyzes the breakdown of pectin by an elimination reaction, which leads to the formation of unsaturated galacturonates and methyl galacturonates [48].

Lytic polysaccharide monooxygenases (LPMOs) play essential roles in the bioconversion of recalcitrant polysaccharides such as chitin and cellulose [51–53], which is required for biofuel production. They belong to a group of copper-dependent oxygenase that split polysaccharides into monomeric units [54]. LPMOs were first identified in fungi as far back as 1990 during a bioprospecting study, as a cellulose-degrading hydrolase [55–57]. It was initially placed in the glycoside hydrolase family GH61 and CBM33 in the CAZy database of carbohydrate-active enzymes [58], but the name later changed to polysaccharide monooxygenases (PMOs) in 2011 and subsequently LPMO [57, 59–63]. *Thermoascus aurantiacus*, a cellulase in the GH61 family, was first confirmed as having the ability to hydrolyze lignocellulosic biomass leading to the re-classification from GH61 to AA9 family, and CBM33 to AA10 family of LPMOs [54, 61, 64]. Some enzymes have recently been found to degrade hemicellulose substrates in addition to cello-oligosaccharide substrates [54, 65]. In the case of bacterial enzymes in the AA10 family, they act on cellulose and chitin unlike the AA9 family, which acts on cellulose and hemicellulose. LPMOs of the AA9 family were identified in several strains of fungi, including *T. terrestris, Neurospora crassa, Podospora anserine, Aspergillus nidulans, Myceliophthora thermophila,* and *Sporotrichum pulyverolentum* [54, 64, 66–68].

The catalytic action of LPMO from *Aspergillus nidulans* on the oxidative degradation of different types of polysaccharides was studied by Jagadeeswaran et al. [68]. An AA9 LPMO in *A. nidulans* the AN3046 was found to be very active in the degradation of cellulose and hemicellulose xyloglucan, which also had a synergistic effect with some sorghum stover degrading hydrolases as it resulted in approximately 1.25-fold increase in glucose yield compared to sole treatment with endoglucanase EglA [68]. In another study, an LPMO from *Aspergillus niger* AnLPMO15g, enhanced the catalytic ability of cellulase in the degradation of Avicel® and straw, which resulted in an increase in the reducing sugar yield by 1.93 and 2.31 times more than that obtained from using only cellulase [54]. The AnLPMO15g enzyme had more activity on Avicel® than other substrates producing the highest yield compared to the other substrates, indicating a high activity of oxidative cleavage on β-1,6 glycosidic bonds [54]. Since the AnLPMO15g also yielded reducing sugars with xylan as a substrate, it has the potential to act not only on β-1,6 glycosidic bonds found in cellulose but also the β-1,4 xylosidic bonds in xylan. From the study of Du et al. [54], we can understand clearly that the synergistic effect of the LPMOs such as AnLPMO15g in increasing the yield of reducing sugars, is dependent on the type of substrate used. This is an important factor to be taken into consideration when selecting LPMOs for biofuel production. Extensive reviews on the mechanism of action of LPMOs in lignocellulosic biomass degradation have been recently published [51, 54, 57, 64].

### Fermentation of lignocellulosic biomass for biofuel production

Bioconversion of lignocellulosic biomass feedstock to biofuel is gaining significant prominence globally. Bioconversion of lignocellulose to biofuels entails four main processes: (1) the pretreatment process, which can be physical, chemical, or both that involves depolymerizing the biomass partially, (2) the enzymatic process, which involves cleaving polysaccharides to simple sugars by the actions of glycan-depolymerizing enzymes, (3) the fermentation process which involves converting the sugars to bioethanol, and (4) lastly, the distillation process which involves separating the bioethanol from water and residual solids [69].

The pretreatment of lignocellulosic biomass and reduction in the cost of the hydrolysis step are major drawbacks to the improvement of biofuel production [70, 71]. The pretreatment process could either be by physical, chemical, or biological means. However, no single method is efficient. Thus, a combination of chemical and biological treatment is often used to obtain higher yields of reducing sugar. Compared to other pretreatment processes, biological pretreatment is found to be less expensive and operates under a mild condition. It requires the use of microorganisms to effectively degrade lignocellulosic feedstocks using different metabolic pathways, directed by the actions of hydrolyzing enzymes such as manganese peroxidases (EC 1.11.1.13), lignin peroxidases (EC 1.11.1.14), and laccases or white-rot fungi [72–75], with the removal of lignin.

Actinobacteria are an essential group of microorganisms known for their ability to degrade several substrates and synthesize products of economic value from the bioconversion of agricultural and urban wastes and the biotransformation of organic compounds [75]. Members of this group have been implicated in the biosynthesis of a wide array of useful enzymes such as xylanases [75–77], chitinases [75, 78], cellulases [75, 79], laccases [80, 81], and proteases [75, 82] required for the degradation of lignocelluloses, lignin, cellulose as well as plant residues [75, 83–85]. *Streptomyces* spp. are recognized for their metabolic potentials, especially in the biosynthesis of antibiotics, and their capability to degrade a range of distinct compounds such as lignocellulose, keratin, pectin, xylan, cellulose, lignin, chitin as well as styrene [5, 75, 86]. Adegboye et al. [5] identified two new strains of *Streptomyces* (NWU339 and NWU49) isolated from maize rhizosphere soil, with the ability to utilize starch, xylan, and cellulose as substrates which could be used for biofuel production. The hydrolytic enzymes synthesized by *Streptomyces fulvissimus* CKS7 (amylase, cellulases (Carboxymethyl cellulase-CMCase and Avicelase), pectinase and xylanase [87], effectively hydrolyzed horsetail waste resulting in maximum yield of bioethanol from the fermentation process with *Saccharomyces cerevisiae* [87].

Apart from microbes, cellulolytic enzymes can be obtained from insects such as termites. In recent times, wood-eating termites have received much interest as a valuable source of cellulolytic enzymes, which are useful for biofuel production [88]. The *Cohnella* genus of bacteria is known for its high cellulolytic activities in different habitats, including the gut of termites. It was recently confirmed as been part of the cellulolytic microbiome associated with wood-eating termites and was identified in the intestinal tracts of three Neotropical termites *Nasutitermes aquilinus, N. corniger,* and *Cortaritermes fulviceps *[88]*.* These wood-eating termites are essential as they have an efficient lignocellulolytic digestion system that could be harnessed for the advancement of the current bioconversion mechanisms of lignocellulosic biomass for the production of useful bioproducts [89]. β-glucosidases have been reported mainly in the salivary glands and midgut of most *Nasutitermes* sp., and xylanases belonging to the GH10 and GH11 families isolated and recombinantly expressed from *Nasutitermes* sp. as well as *Globitermes brachycerastes* bacterial symbionts, respectively [90, 91]. Thus, termites should be considered as biological models for bioprocessing of cellulosic biomass [91]. Moreover, there is a need to utilize the advancement in omic technologies to identify key genes required for cellulolytic enzyme synthesis, which could be utilized for bioengineering of useful microbial strains for biofuel production.

**Fig. 3:**
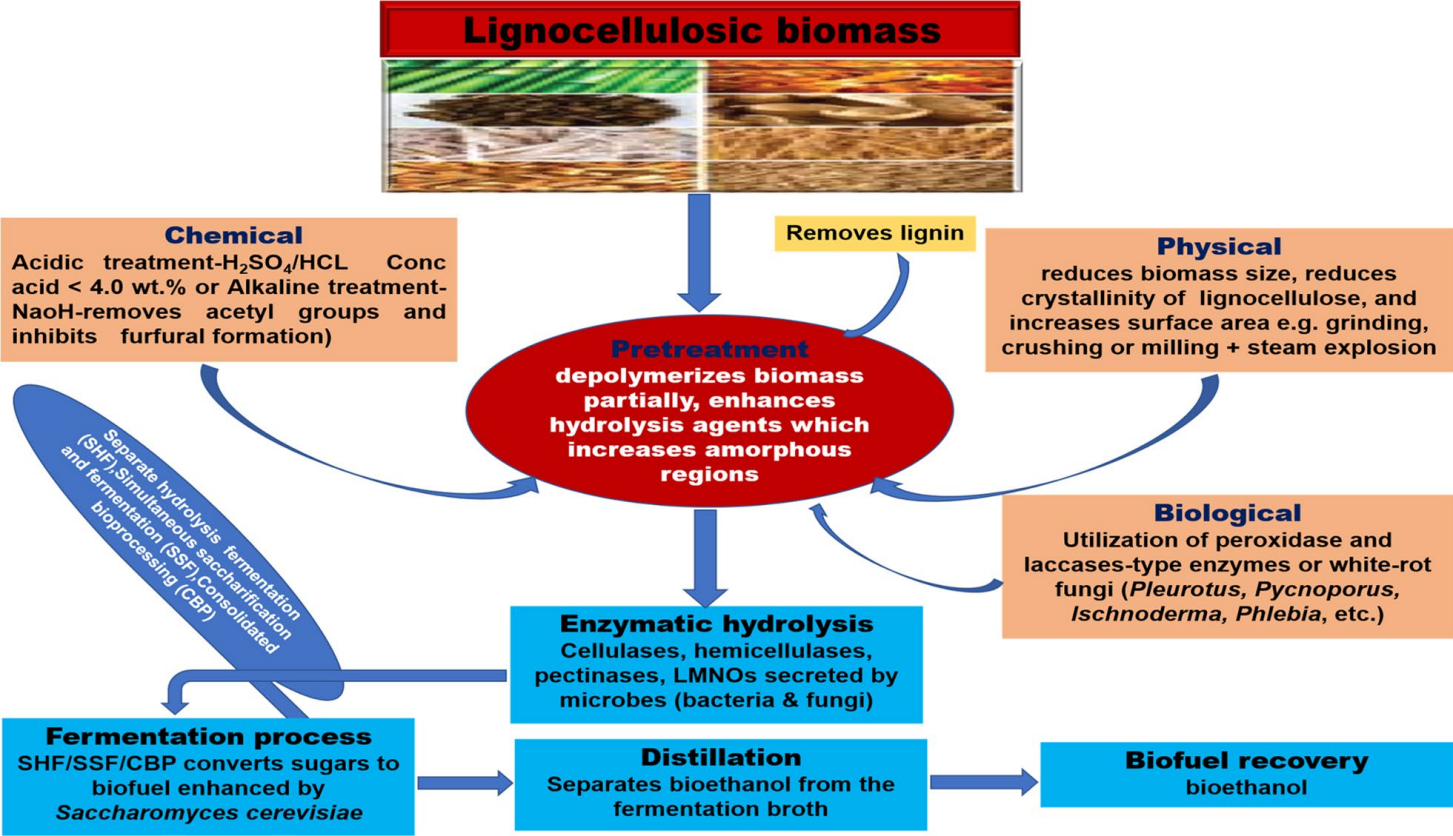
Flowchart of bioethanol production from lignocellulosic biomass

Although biological pretreatment is regarded as the most effective delignification approach, factors such as particle size, moisture content, biomass type as well as the nature of the microorganism could hamper the pretreatment process [92]. Moreover, the biological pretreatment of lignocellulosic biomass is relatively slow and can take several days before it is fully hydrolyzed. The chemical pretreatment offers many potentials as it increases the porosity of the biomass as well as solid separation [71, 93]. Nevertheless, due to the harmful effect of the chemicals used for pretreatment on the environment when been disposed of and the difficulties involved in recycling, the chemical pretreatment method is not frequently used alone [93–95]. The chemical pretreatment could be either acidic or alkaline. The flowchart for the production of bioethanol is presented (Fig. 3).

The use of acidic treatment is not recommended due to its toxicity to the microorganisms used in the fermentation process, and possibly corrosion to the fermentation vessels. Nevertheless, some researchers have reported appreciable yield of reducing sugars at concentrations < 4.0 wt % and temperatures of 120–210 °C. Lu et al. [96] obtained a yield of 77% xylose and 8.4% glucose from the enzymatic degradation of corn stover following pretreatment with H_2_SO_4_ (2%) at 120 °C for just 43 min, which was regarded as the optimum conditions for pretreatment [96, 97]. Whereas, Bhandari et al. [98] obtained a higher yield of xylose and glucose (78.7% and 18.7%) with 1.47 wt % H_2_SO_4_ at 155 °C for 31 min, and 78.1% xylose and 14.5% glucose yield at 182 °C for 36 min, respectively [98]. In a related study using olive tree biomass, pretreatment with 1% H_2_SO_4_ at 180 °C gave a maximum overall yield of 75% of total sugar [99]. From the foregoing, it is evident that the yield obtained from enzymatic saccharification after pretreatment with the acid varied in terms of three factors, namely concentration of the acid, time of exposure, and temperature used with good yield obtained at a temperature of 120–182 °C. The use of sulfuric acid in the pretreatment step aids in the solubilization of hemicellulose into monomers which increases the digestibility of cellulose by hydrolytic enzymes [92, 100].

Alkaline pretreatment of lignocellulosic biomass is usually performed with NaOH at low temperature and pressure. It is most preferred to acidic pretreatment due to a reduction in the loss of carbohydrates during hydrolysis [92, 97, 101]. It is known to expel acetyl groups, thereby enhancing hydrolysis in the subsequent step and inhibit furfural formation [97], subsequently removing lignin and hemicellulose. Alkaline pretreatment is most suitable for agricultural wastes like wheat straw which enhances the digestibility of cellulose without degrading both hemicellulose and acid treatment [92]. The use of alkali pretreatment along with microbial hydrolysis of the pretreated biomass [32], has recently been shown to preserve polysaccharides while significantly removing lignin as was evident in wheat straw pretreated with 10% NaOH which resulted in 72.67% yield of cellulose and removal of 69.5% lignin [32, 102]. Moreover, total reducing sugars (83.68%) were recovered after alkaline pretreatment of the wheat straw and microbial hydrolysis of cellulose and hemicellulose [32]. In many cases, the combination of biological and chemical pretreatments is more effective and requires less rigorous pretreatment conditions to efficiently hydrolyze the feedstock [103].

After the hydrolysis of the feedstock by the various enzymes [cellulase (endoglucanase, exoglucanase, and β glucosidase), hemicellulase (β-1, 4- xylanase, β-1,4 xylosidase), pectinases (hydrolase-endopolygalacturonase and exopolygalacturonase, Lyase-polygalacturonate Lyase and Polymethylgalacturonate Lyase, esterase and lytic polysaccharide monooxygenase)] as discussed above, the carbohydrate components (cellulose and hemicellulose) become fermentable. This attribute makes lignocellulosic biomass an attractive feedstock for biofuel production [104]. Through the process of fermentation, the hydrosylate obtained after removal of lignin in the pretreatment stage and hydrolysis is converted to biofuel. Yeast (*Saccharomyces cerevisiae*) is the preferred choice of microorganism for fermentation of sugars to bioethanol due to its ability to tolerate high ethanol concentrations and inhibitors produced during the fermentation process.

The fermentation process for ethanol production could occur in three ways; it could be run separately immediately after the hydrolysis step, which is regarded as separate hydrolysis and fermentation (SHF) [104], or the saccharification takes place simultaneously with fermentation, described as simultaneous saccharification and fermentation (SSF), or the production of cellulase, as well as the enzymatic reaction and fermentation, occur simultaneously in the same bioreactor, a process called consolidated bioprocessing (CBP). These fermentation methods are frequently used for bioethanol production, with increased yields obtained. However, each has its advantages and drawbacks.

The SHF provides optimum working conditions for hydrolysis and fermentation, which occurs in separate vessels at different temperatures and enables the recycling of the yeast used in fermentation [92]. Besides, it permits a continuous run of the fermentation process. Hydrolysis by cellulase occurs efficiently at a temperature range of 45–50 °C, while fermentation by microbes occurs at temperatures of 30–37 °C, resulting in ethanol production [92, 104–106]. Nevertheless, the end products (glucose and cellobiose) inhibit the activity of the cellulase enzyme and require more time to run the process [92, 97]. β-glucosidase is inhibited by glucose, which elevates the level of cellobiose. Cellobiose subsequently inhibits cellulase, thereby reducing its efficiency [106, 107]. Furthermore, SHF is a two-step process that incurs additional cost, and it is time-consuming.

The SSF is the most preferred fermentation method for bioethanol production from lignocellulose as the processes of enzymatic hydrolysis and fermentation occurs within the same bioreactor, thereby reducing the cost of production, improving ethanol yield, as well as reducing the risks of contamination and enzyme inhibition by the end products of hydrolysis [92, 108]. This is made possible because before the inhibitory concentrations of the end products are reached, the glucose and cellobiose produced are simultaneously fermented to high-energy–density ethanol molecules [109]. Besides, different lignocellulosic substrates could be used under various pretreatment conditions that result in increased product yield within a short period [109]. SSF can be conducted using fermenting thermophilic bacterial strains and yeast cells such as *Candida acidothermophilum* and *Kluyveromyces marxianus* without compromising the optimal temperature of hydrolysis [109].

Mihajlovski et al. [87] obtained a high yield of bioethanol from the SSF of rye bran using crude enzymes produced by *Streptomyces fulvissimus* CKS7*.* A maximum reducing sugar yield of 2.55 mg ml^−1^ was obtained using horsetail as substrate after 72 h of hydrolysis followed by fermentation with waste brewer’s yeast *S. cerevisia*e [87].

Despite the maximal yield of ethanol obtained through SSF of hexoses, there is a drawback in the fermentation of pentoses, which are omitted when only a hexose fermenting strain such as *S. cerevisiae* is used that makes it necessary to use a pentose-fermenting strain in a separate bioreactor after pretreatment, to complete the fermentation process [109]. Both SHF and SSF require the introduction of enzymes for hydrolysis.

In the case of CBP fermentation, a microbial consortia biocatalyst strategy is used by combining a cellulolytic strain capable of hydrolyzing hemicellulosic biomass to fermentable sugars, and a second strain that makes use of the cellulosic sugars for its growth and converts them to biofuel products during the fermentation process using its natural or engineered metabolic pathways [110]. Unlike the SHF and SSF, in the CBP, the three stages of enzyme synthesis, hydrolysis of lignocellulosic biomass, and fermentation occur concurrently in the same bioreactor. This reduces the cost of biofuel production as a result of less complicated feedstock processing, less energy expended, and higher conversion efficiencies [111]. However, before the CBP approach can be used, it requires microorganisms capable of producing a functional cellulase system while generating ethanol at high returns and concentrations [112]. In this regard, bacteria such as *Clostridium thermocellum* and fungi such as *Neurospora crassa, Fusarium oxysporum, S. cerevisiae,* and *Paecilomyces sp. *[104]*,* come in handy for biofuel production (ethanol and butanol) using CBP approach [113–115].

After the fermentation process, the product obtained needs to undergo the purification process and distillation to separate the bioethanol from the fermentation broth to obtain the final pure bioethanol separating it from the fermentation broth. The amount of bioethanol produced from the fermentation process depends mainly on the number of sugars produced during pretreatment and for hydrolysis efficiency [116, 117].

The fermentation process for biofuel production is often expensive; hence, effort needs to be made to seek alternative means of making the process cost-effective. One such way is to reduce production cost through high solids loading as a result of reduced water uptake and downstream processing cost, and this ultimately helps to reduce environmental pollution [118]. Some difficulties arise because of the high loading of lignocellulose solids, for instance, inhibition of enzymes by end products. This can be overcome through the application of fed-batch processes [108, 119]. The success of this kind of process can be measured by the total yield of bioethanol produced (volume of ethanol produced per dry weight of raw material) and the level of ethanol concentration in the fermentation batch [119].

### Strains for biofuel production

The successful production of biofuel from lignocellulosic biomass depends mainly on finding and exploiting a suitable microorganism for the whole fermentation process [120]. The ideal strain for biofuel production should be able to completely utilize the pentose-rich and hexose containing sugars produced from lignocellulosic biomass feedstock, and that can survive the inhibitory compounds that are generated during the pretreatment step. Most of the organisms employed for fermentation are not able to utilize pentose sugars, and those that can ferment it are inhibited by end products and by-product formation [121, 122].

*Saccharomyces cerevisiae* and *Zymomonas mobilis* are the best-known alcohol fermenting microbes with the ability to ferment hexose sugars and sucrose into ethanol but are inhibited by end products [123, 124]. Moreover, pentose-fermenting organisms, *Pichia stipitis*, *Candida shehatae,* and *Pachysolen tannophilus* [125], are also inhibited by end products [123, 126]. Even though filamentous fungi can withstand inhibitory compounds, their high generation time and lower yields and productivities make them unattractive candidates for biofuel production [125]. Thus, a microorganism that is inhibited by end products, and that also takes more time to hydrolyze the lignocellulosic biomass is not appropriate for industrial-scale production of biofuel [15].

The ideal strain can either be a natural cellulolytic biofuel-producing microbe or an engineered industrial strain conferred with the gene(s) to produce biofuel [120]. The ideal strain needs some attributes to use high amounts of substrates such as the ability to attain high cell mass growth and biofuel production rates in biomass-derived hydrolysates [127], the ability to use a wide range of pentose and hexose sugars withstand high temperatures and low pH [127, 128], as well as to exhibit good tolerance to inhibitors and end products. This strain should also have high metabolic fluxes and biosynthesize single fermentation products for sugar transport through fast and deregulated pathways. It is easier and more economical to operate and control a bioreactor at extremely high temperatures. Operating high temperatures also advances reaction rates, viscosities of culture broth, and decreases the risk of contamination during production. Also, the ability to adapt to lower pH can help lessen the rate of contamination from many interfering microbes [128]. All these attributes must be put into consideration by the metabolic engineers when trying to develop the most suitable microbe for large scale production of biofuel.

### High substrate utilization ability

The model microbe used for biofuel production must be able to hydrolyze lignocellulosic biomass substrate and produce the desired end products at a high amount under industrial conditions. For a strain to use a high amount of lignocellulosic sugars, several attributes must be put into perspective. Primarily, the strain must be able to achieve a high cell mass growth index in a short time and recovery of biofuel from the biomass-derived hydrolysates that could contain inhibitory substances such as aromatic compounds, acetate, and aldehydes [128]. Furthermore, the ability to use a wide range of sugars such as pentose, hexose, and disaccharides is of great importance in biofuel production. Finding microbes that can achieve such desirable traits can be either through screening or incorporation of such genes [129, 130].

Several groups have reported the use of *Saccharomyces cerevisiae* and *Escherichia coli* as engineered industrial strains [131–135]. Most industrial strains do not metabolize other sugars in the presence of glucose because of carbon catabolite repression [136–138], which serves as a limitation for their use with substrates such as lignocellulose. Sievert et al. [137] reported the solution to this restriction by engineering *E. coli* strains with a point mutation in a transcriptional activator for catabolic operons, thus leading to catabolic activation independence of the catabolite repression control [138]. Alternatively, introducing transporters together with gene expression encoding for the utilization of other sugars have also alleviated glucose repression and facilitated co-fermentation [139] This approach had been proven by engineering *E. coli* and yeasts to co-metabolize several combinations of sugars [136].

### Good tolerance to inhibitors and end products

One crucial issue that has to be overcome to reach an optimum yield of biofuel production is to enhance tolerance of strains to inhibitory compounds, metabolic intermediates, and the desired end products [140]. Examples of toxic compounds present in lignocellulosic hydrolysate include furan derivatives, weak organic acids, and phenols. As microbial cell growth is important to increase biofuel production, engineering robust strains with high tolerance to inhibitors is imperative. During microbial fermentation for biofuel production, the accumulation of end products and by-products can negatively affect the growth of microbes, thus impeding the level of production. Consequently, it is imperative to advance tolerance to inhibitors when engineering strains for biofuel production [140, 141].

The engineering of strains to have special features such as high tolerance level to inhibitory compounds is crucial in accomplishing high productivity and sustainable industry. This can be achieved through rational and evolutionary engineering [142]. Rational engineering entails direct manipulation of known genetic components such as transcriptional regulators, transporters, and pathway enzymes identified [140], because of previous knowledge of the mechanisms of toxicity and tolerance. Whilst evolutionary engineering involves indirect manipulation through adaptation or mutagenesis resulting in the evolution of strains, high-throughput screening is used in the selection of strains with high tolerance level [143].

The factors involved in producing enhanced tolerance strains are explicated through using systematic biological techniques, endowing parental strains, and other native strains with the desired tolerance phenotype by genetic exploitations of uncovered target factors [140]. To demonstrate the potential for improving tolerance of microbial strains, Atsumi et al. [144] serially transferred cultures of *E. coli* for enrichment in isobutanol to obtain tolerant strains. After five rounds of culture transfer, an engineered (mutant) strain displayed 2.0% (w/v) isobutanol tolerance, whereas the wild type *E.coli* strain (JCL16) lacked tolerance since 1.5% (w/v) isobutanol inhibited its activity [144]. Furthermore, tolerance improvement strategies such as global transcription machinery engineering can be used [144]. Most metabolic engineering research involving biofuel production emphasizes enhancing the catalytic effectiveness of a sole reaction. Alper et al. [143] developed a global transcription machinery engineering (gTME) system to enhance glucose/ethanol tolerance in *S. cerevisiae*. This method entailed reprogramming gene transcription to obtain cellular phenotypes vital for the technological approach. Transcription factor Spt15p was subjected to mutation which brought about a rise in tolerance and more effective glucose conversion to bioethanol. The resulting phenotype was from the combination of three different mutations in the Spt15 gene containing Phe177Ser, Tyr195His, and Lys218Arg [143]. Therefore, gTME can provide a channel to complex phenotypic traits that are not readily accessible by conventional approaches. This was recently confirmed by El-Rotail et al. [145] who designed *SPT15* mutagenesis library of *Saccharomyces cerevisiae* using the gTME approach. The authors obtained a novel mutant of *S. cerevisiae* with a higher tolerance to ethanol stress when treated with 3% MnCl_2_ in place of the widely used mixture of error-prone (Ep-PCR) reaction with MgCl_2._ and yielded the highest ethanol production.

### High metabolic fluxes

One of the constraints experienced by engineered microbial cell factories includes metabolic imbalance as a result of nutrients depletion, metabolite accumulation, evolutionary pressure, genetic instability, or other stress factors [146]. It is of tremendous importance when developing the model strain to be equipped with a sensor-regulator system that will allow the cell to adjust metabolically in response to the surrounding changes [146]. Metabolic fluxes have been greatly manipulated by metabolic engineers to improve the model strain abilities in the production of biofuel [147, 148].

Approaches such as fed-batch cultures, mutagenesis, and optimal control of the metabolic pathways have been developed to cope with the balance between cell density and product formation and to enhance the cost-effectiveness of industrial fermentation [146]. With the aid of metabolite-responsive transcriptional factors [146], metabolic engineers can now engineer cell factories to realize self-adaptation for biotechnological applications. This could be achieved by revamping the transcriptional regulatory networks and aiding the cell to independently regulate pathway expression and modify the metabolic activity to the changing environment [139, 146, 149, 150]. Alternatively, the dynamic control theory could be used to maximize pathway efficiency [146, 151]. Xu et al. [152] used this approach by engineering naturally occurring transcriptional regulator FapR to control the fatty acids biosynthetic pathway in *E. coli*. Fatty acid production is significantly developed by optimum control of gene expression resulting in balanced metabolism between the growth of cells and the formation of products. Application of metabolic control enables the engineered strain to dynamically control pathway expression and balanced the metabolic activity of key enzymes based on the intracellular level activities.

Insight into the complex regulation of metabolic fluxes can be known through the function of three factors in a given biochemical reaction namely (i) enzymatic activity of the catalyzing enzyme (ii) characteristics of the enzyme (iii) the effects of substrates and metabolites on the enzymatic activity. The enzymatic activity exhibited by a strain is due to gene expression, translation, and post-translational protein modifications. The enzyme traits are usually specific for a given biological system under research. However, in situations where the heterologous enzymes are introduced to redirect metabolic fluxes, it becomes imperative to study the traits of the heterologous enzyme in comparison to other enzymes having interaction with the same metabolite pools [146]. Feedback regulation was imposed on the system by the concentration of metabolites and properties of the enzymes which serve important functions in the metabolic fluxes [153].

### Bioprospecting for native strains with the gene of interest

Extensive research has shown that several microbes belonging to the class fungi, yeast, and bacteria can exhibit cellulolytic activity [24, 154]. Today, the process of bioethanol production exploits cellulolytic enzymes from microbes with some strains having established industrial applications, a high conversion rate of glucose to ethanol and tolerance to end products, and other inhibitory compounds [6, 155]. However, during their evolution, some of these organisms have not been exposed to the conditions obtainable under industrial settings that typically arise in the industrial processing of feedstock to biofuel.

Screening for a particular trait is one of the most effective ways of discovering novel enzymes applicable to the industry [156]. Native strains produce diverse extracellular and intracellular enzymes naturally that could exhibit activities of industrial importance. One of the common methods used for finding these strains is bioprospecting. Bioprospecting involves screening native strains isolated from diverse sources for novel and functional enzymes which might be relevant. These microorganisms are isolated from different environments and are explored for their ability to utilize certain substrates for biofuel production [157]. Consequently, the selection of the best candidate is based on the high production of the desired end products. Another approach is probing the genome contents of environmental samples through metagenomics. The use of probes and primers specific to target certain gene(s) of industrial importance [158, 159].

The main drawback of this approach is that it is qualitative: the metabolic perspective cannot be quantified because isolation and culturing of the microbe cannot be achieved [159]. Analyzing the genetic make-up of the strains helps in the prospecting of potential microorganisms very quickly, which facilitates the evaluation of the proteome of the microbes and to determine if the isolate possesses the gene(s) of interest. Besides, the bioprospection of genes of interest by metagenomic strategies allows the identification of uncultured microorganisms [160, 161].

Bioprospecting contributes significantly to the advancement of biofuel production. For instance, the isolation of extremophiles from exotic locations leading to successive extraction of interesting enzymes. Using such microorganisms is advantageous in the sense that they can produce special enzymes that can withstand different industrial conditions such as high temperature, salinity, and pressure [162]. One of the major benefits of exploiting enzymes from hyperthermophiles is the reduction of contaminants from the operating system. Besides, high temperatures also result in very low viscosity and increase the solubility of substrates, ultimately leading to high yields as a result of favorable displacement of the equilibrium in endothermic reactions [159]. The successful use of native strains to produce biofuel entails having a better insight into their physiology under various conditions and subsequent strain improvements.

### Process of fermentation using metabolic engineered strains

The lignocellulosic biomass can be fermented by several microbes [116, 126] but the complete utilization of lignocellulosic biomass for the production of biofuel is impeded by the lack of model strains that could effectively degrade both pentose and hexose sugars to glucose [25]. An ideal industrial strain sustainable for commercial production of biofuel should use a wide variety of substrates, produce a higher yield of end products, tolerate high levels of end and by-products, and high temperature, should be able to withstand inhibitory compounds and have high cellulolytic activity [163–165]. Moreover, microbial hosts should exhibit sturdiness against stresses and toxic chemicals, and scale-up, and actual commercialization of advanced biofuels.

Metabolic engineering has been used to modify native strains increasing the production of biofuels. The production of biofuel has been developed from a variety of biomass feedstocks (from starch-based to lignocellulose) by engineering or developing the metabolic pathways of diverse microbial hosts [166–168]. The concept of metabolic engineering with the aid of recombinant DNA technology, brought about the improvement of biosynthesis of desired products by the exploitation of biosynthetic pathways, transport systems, and regulatory functions of the cell [169]. Genetic engineering employed the use of classical mutagenesis and selection and recombinant methods for the over-expression of the desired end product during the process of fermentation associated with pathways. Strains are engineered in the laboratory to make them tolerant to high concentrations of end products and other inhibitory substances by removing the normal regulatory genes and enzymes associated with the metabolic pathway. The ultimate goal is to develop a robust fermentation process that facilitates the high production of the desired product(s) with little or no bottleneck.

For metabolic engineering of the strain to be regarded as being successful, the whole process must be cost-effective on a large scale. To achieve this, researchers had to develop novel techniques such as whole-genome sequencing, bioinformatics, systems biology, proteomics, and metabolomics. All these techniques have significantly assisted researchers in enhancing the applications of metabolic engineering over the past years. These have helped in developing novel engineered strains that can carry out high-throughput performance using renewable feedstocks such as lignocellulose, rationalizing production cost even more.

Production of higher octane hydrocarbons which are substitutes to ethanol such as 1-butanol, isobutanol, and isopentanol, with improved fuel qualities, are biosynthesized through engineering fermentative pathways, non-fermentative keto acid pathways, and isoprenoid pathways [170–172]. Amongst higher alcohols, fatty-acid-derived and isoprenoids-derived biofuel from microorganisms have also been suggested as superior fuel alternates. Several native isolates and their metabolic pathways have been investigated comprehensively to improve yield, titer, productivity and to reduce the cost of production using various strategies [173–178]. The application of genetic and metabolic engineering approaches have led to significant advancement by improving existing applications and also opening up new possibilities [179, 180]. These approaches have improved the physiology of the potential producers of biofuel, enabling high and cost-effective production. Due to different mechanisms of action within the hosts, it would be difficult to ascertain a conventional approach that will work for the different types of biofuels obtained from diverse metabolic pathways [181, 182]. Heavy reliance on fossil fuel and the effect of global warming can be reduced by providing environmental-friendly energy to power automobiles and other industrial appliances.

Most of these difficulties can be addressed by tailoring the redesigned metabolic system of each microorganism to suit the end product in other to advance yield and productivity, and ultimately reduce operating cost [180]. A successful outcome from genetic engineering can translate to effective land use and biodiversity. For instance, the maximum production of biofuel from lignocellulosic biomass feedstock corresponds to an equal saving in land usage because fewer raw materials are needed. Metabolic engineering of microorganisms to make use of various feedstocks effectively can as well sustain native flora by decreasing the need for requiring non-native plants [183].

Metabolic pathways can be engineered to biosynthesize new products that can replace fossil fuel including long octane numbers short-chain, branched-chain, and cyclic alcohols, alkanes, alkenes, esters, and aromatics compounds. Understanding the need for superior fuel is of importance to develop strains that will produce alternate biofuel with useful applications [19]. One of the major shortcomings in these processes is how to enhance carbon assimilation in the metabolic pathways and then control the fluxes of these pathways to biosynthesize product(s) interest either by natural or engineered pathways [183]. Many of these desired products are sought after because of their outstanding qualities for more specialized applications. Nevertheless, while some of these compounds or their precursors can be biosynthesized from diverse metabolic pathways that exist naturally in microorganisms, these pathways often need to be optimized or redesigned to advance effectiveness. Moreover, practical or theoretical yields are calculated based on biosynthetic pathways and levels of productivity. The unavailability of genetic engineering platforms for native isolates, coupled with challenges in the optimization of the metabolic pathways, and balancing the redox state in engineered strains are major drawbacks to the development of low-cost industrial processes for the conversion of lignocellulosic biomass feedstocks into biofuel [19, 103]. The engineering of biosynthesis pathways in native strains can bring about an increase in biofuel formation. For instance, the engineering of electron metabolism in *Clostridium thermocellum* increased bioethanol production [184]. Several metabolic engineered strains of microbes have been used successfully for biofuel production (Table 2).

**Table 2:** Metabolic engineered strains of microorganisms used for biofuel production

Organisms	Product	Pathway	Substrate	References
*Corynebacterium glutamicum*	3-Hydroxypropic acid	Glycerol	Glucose, xylose	[185]
*Clostridium autoethanogenum*	Ethanol	Ferredoxin oxidoreductase	Synthetic medium	[186]
*Synechocystis* sp.	Isobutanol	Ehrlich	Glucose	[187]
*S. elongates*	1,3-Propanediol	Synthetic metabolic pathway	Synthetic medium	[188]
*E. coli*	Fatty alcohol	Fatty acyl-ACP reductase-dependent	Synthetic medium	[189]
*Saccharomyces cerevisiae*	2,3-Butanediol	Butanediol biosynthetic	Glucose, galactose	[190]
*Klebsiella pneumoniae*	2-Butanol	Meso-2,3-butanediol synthesis	Glucose	[191]
*C. cellulolyticum*	n-Butanol	CoA-dependent	Cellulose	[15]
*Thermoanaerobacterium saccharolyticum*	n-Butanol	n-butanol	Xylose	[232]
*Enterobacter cloacae*	2,3-Butanediol	Pentose phosphate	Lignocellulose	[192]
*Methylobacterium extorqens*	1-Butanol	Ethyl malonyl-CoA	Ethylamine	[193]
*C. cellulovorans*	Ethanol, n-Butanol	Fatty acyl-ACP reductase-dependent	Cellulose	[194]
*Caldicellulosiruptor*	Hydrogen	Glycolytic	Lignocellulose	[195]
*Bescii*				
*S. cerevisiae*	Isobutanol	Embden-Meyerhof	Synthetic medium	[196]
*S. cerevisiae*	n-Butanol	Clostridial acetoacetyl-CoA-derived pathway	Synthetic minimal	[197]
*S. cerevisiae* strain XUSAE57	Ethanol	Xylose‐isomerase pathway	Xylose and Glucose	[198]
*Clostridium Tyrobutyricum*	n-Butanol	Xylose metabolic pathway	Glucose and Xylose	[199]
*Clostridium acetobutylicum* and *Saccharomyces cerevisiae*	n-Butanol	Clostridial acetoacetyl-CoA-derived pathway	Glucose, corn, starch, and corn stover	[200, 201]
*Clostridium thermocellum* and *Thermoanaerobacterium saccharolyticum*	Ethanol	Embden-Meyerhof	Cellulose	[202]
*E. coli*	1-butanol,	2-keto acid degradation pathway	Glucose	[144]
	2-methyl-1-butanol, 3-methyl-1-butanol, 2-phenylethanol			

**Fig. 4:**
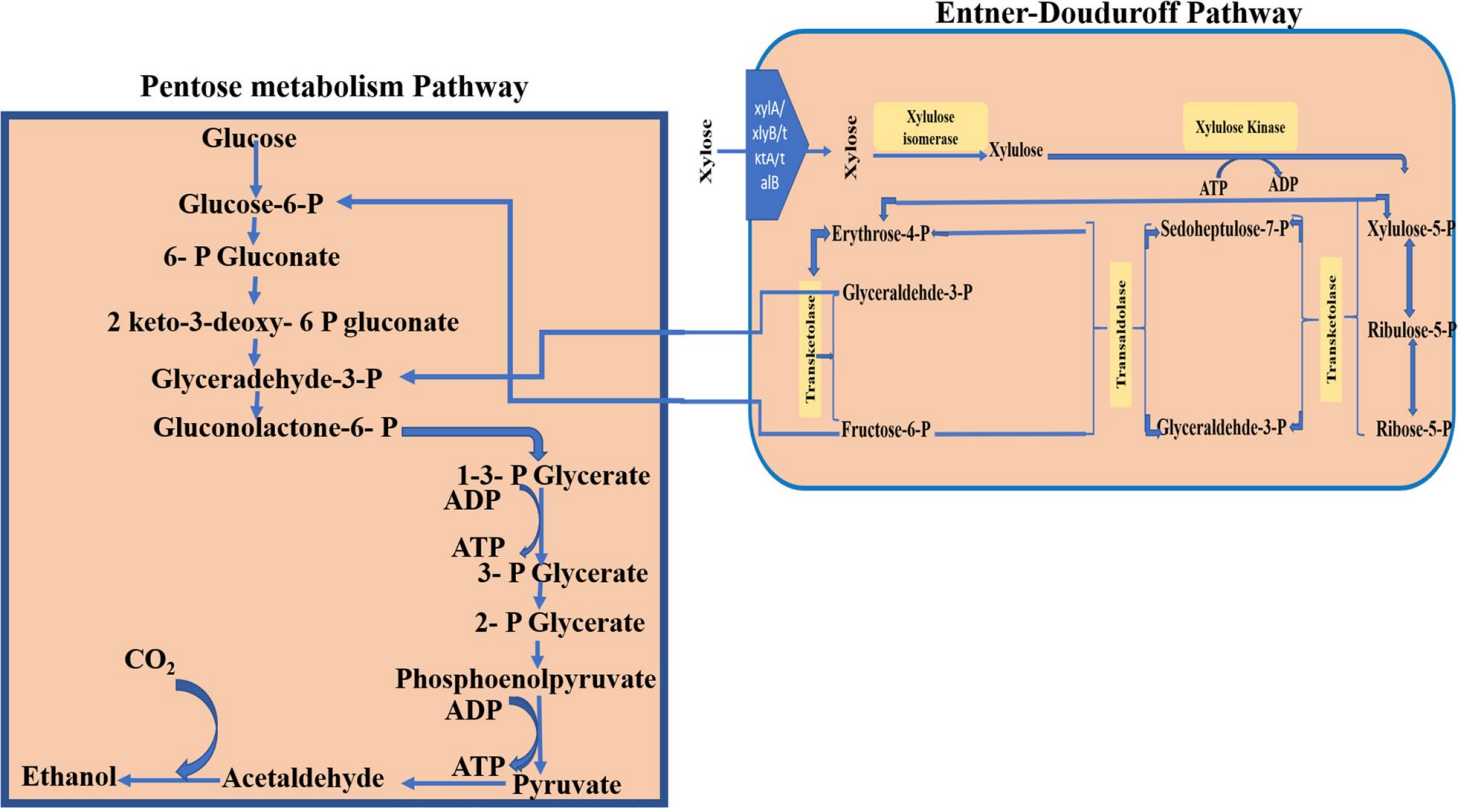
Pentose metabolism and Entner–Duoduroff pathways in transformed *Zymomonas mobilis*

Through the manipulation of the genetics and metabolic pathways of microorganism, scientists have been able to enhance the production of specific metabolites which have been used successfully in the production of biofuel and other products. For instance, *Zymomonas mobilis* a well-known indigenous producer of bioethanol can only efficiently utilize hexose sugar (glucose) converting it to bioethanol compared to *Saccharomyces cerevisiae* but not from pentose sugars. This is a major drawback in its utilization for biofuel production from lignocellulose biomasses which are rich in pentose sugars. *Zymomonas mobilis* lacks a complete pentose phosphate pathway due to the absence of transaldolase activity [203] *Z. mobilis* uses the Entner–Doudoroff (ED) pathway which is more efficient than the Embden–Meyerhof–Parnas (EMP) pathway utilized by *S. cerevisiae* with less use of ATP [204] To circumvent this challenge, a metabolic engineering approach was used to enable *Z. mobilis* to utilize pentose sugar by introduction and expression of genes encoding the enzymes xylose isomerase, xylulokinase, transaldolase and transketolase which created a complete metabolic pathway for the conversion of xylose to important intermediates of the EMP pathway (glyceraldehyde -3-phosphate and fructose-6-phosphate) leading to bioethanol production (Fig. 4).

The xylose fermenting strain of *Z. mobilis* was constructed by cloning *Escherichia coli xylA* and *xylB* genes using a potent *Z. mobilis* glyceraldehyde-3-phosphate promoter by PCR-mediated overlap extension [203]. *Z mobilis* was transformed with the xylose assimilation operon obtained but the transformants still could not utilize xylose in the medium due to lack of sufficient transketolase and transaldolase activities [203]. Therefore, an open reading frame that encodes transaldolase *tal* gene on *E. coli* was synthesized using PCR and subsequently subcloned using a *Z mobilis* enolase promoter by PCR- mediated overlap extension. Likewise, a transketolase gene (*tktA*) was synthesized from *E coli* W3110 genomic DNA and subcloned immediately downstream of the transaldolase homolog translation codon giving rise to an operon that encodes the non-oxidative part of the pentose phosphate pathway [203]. The xylose assimilation and pentose phosphate pathway operons constructed were concurrently transferred into *Z mobilis* CP4 using a *Z mobilis* pACYC184. The recombinant *Z mobilis* CP4 (pZB5) grew on xylose containing medium and produced a yield of 86% and 94% ethanol from xylose and glucose respectively [203]. Recently an improved strain of *Z. mobilis* TMY-HFPX was developed containing an operon with several genes *xylA/xlyB/tktA/talB* for the utilization of xylose, the metB/yfdZ operon for lysine and methionine biosynthesis, the thioesterase gene tesA which improves free fatty acid biosynthesis for higher ethanol tolerance, a proton-buffering peptide operon for acid stress tolerance, and a small heat shock protein operon for heat stress tolerance [205]. This strain gave a theoretical yield of 90% ethanol from the utilization of xylose as the carbon source [205] Metabolic engineering has also been used to enhance biofuel production abilities of several other microorganisms, including *Bacillus subtilis* for etha*no*l production*, Clostridium* for butanol production as well as *E.coil*. *B. subtilis* strain BS35 was designed by obstructing the lactate dehydrogenase gene via chromosomal insertion of the *Z. mobilis* pyruvate decarboxylase gene and alcohol dehydrogenase II gene controlled by the ldh native promoter [204, 206]. Although the strain yielded ethanol and butanediol, compared to the wild type, the transformed strain had reduced cell growth and glucose utilization up to 60–70%. Nevertheless, further manipulation of the BS35 to BS36 (BS35 *ΔalsS*) resulted in 89% theoretical yield of ethanol, and by inactivation of *alsS* through chromosomal integration of *E. coli* transhydrogenase gene, a new strain BS37 (BS35 *ΔalsS udhA*^+^) capable of producing 8.9 gL^−1^ ethanol was obtained [206].

*Klebsiella pneumoniae* HR526 a high yielding 2,3-butanediol producing strain was engineered by Chen et al. [191] for the production of 2-butanol. The authors extended the 2,3-butanediol synthesis pathway of the bacterium and introduced diol dehydratases and alcohol dehydrogenases. Optimization of the pathway and engineering of the diol dehydratase via protein engineering resulted in an increased yield of 2-butanol (1030 mg/L). In another study, metabolic engineering of cellulolytic *Clostridium cellulovorans* with the genes for aldehyde/alcohol dehydrogenase (*adhE2*) and an artificial electron carrier methyl viologen (MV) was carried out by Yang et al. [194], in a bid to directly produce ethanol and n-butanol at a higher rate from cellulose. The *adhE2* gene from *Clostridium acetobutylicum* was fully expressed in *C. cellulovoran*s which led to the production of considerable quantities of n-butanol (1.42 g/L) and ethanol (1.60 g/L) from the crystalline cellulose [194]. C. *cellulovorans is a* very useful bacterium for metabolic engineering due to its ability to utilize several substrates and also possesses numerous cellulosomal genes [194, 207]. Recently Bao et al. [208] went a step further to introduce two extra aldehyde/alcohol dehydrogenases encoded by *bdhB, adhE1* in addition to *adhE2* used by Yang et al. [194] from *C. acetobutylicum* into *C. cellulovorans*. Co-expression of either *adhE1* or *adhE2* with *bdhB* genes failed to improve the yield of butanol possibly due to the limiting factor of NADPH in *C. cellulovorans* [194]. The highest yield of butanol was obtained only by the strain overexpressing *adhE2* (4.0 g/L) which was 181.69% times higher than the amount recorded by Yang et al. [194]. Acetic acid is known to disrupt the efficiency of microbes such as *Saccharomyces cerevisiae* during the fermentation process, thereby reducing their bioconversion ability of lignocellulosic biomass to produce biofuel. This recently led Ko et al. [198] to engineer a high xylose utilizing strain of *Saccharomyces cerevisiae* XUSAE57 for enhanced bioethanol production by improving tolerance to acetic acid. This strain was chosen out of the several variants obtained from culturing a previously engineered *S. cerevisiae* strain possessing the xylose-isomerase pathway XUSE developed by PTN Hoang, Ko et al. [209]. This served as the parental strain and was cultured with the adapted XUSAE57 strains in yeast synthetic complete media (YSC) containing 20 g/L xylose, incubated aerobically at 30 °C for 1.5 days for preculture and subsequently in fresh YSC medium containing 20 g/L xylose and 0–5 g/L acetic acid with an initial pH of 5 [198]. This resulted in a twofold increase in ethanol production, in addition to a twofold increase in xylose utilization in contrast to the XUSE strain in the presence of 4 g/L of acetic acid [198]. Besides, the improved XUSAE57 strain till date, has the highest amount ethanol produced from the bioconversion of glucose and xylose from lignocellulose hydrosylate). Metabolic engineering of useful microbial strains will definitely have a great impact on the biofuel industry in the nearest future. This will, however, require identification of useful strains and having a mechanistic understanding of the various metabolic pathways that can be harnessed for better biofuel production.

### Future prospects

Microbial metabolic engineering is not an easy task, especially for the identification of efficient strains, but it is indispensable for the advancement of the biofuel production industries. The important metabolic pathways must be well understood, and the relevant enzymes identified. The limiting regions of pathways are being identified by metabolic engineers and synthetic biologists using different approaches [204, 210–212]. The process of metabolic engineering for enhancing recombinant protein expression keeps on evolving and becoming sophisticated. Industrial microbes have been modified or designed to improve recombinant metabolite productivity while saving time and money [204, 213, 214].

Advanced technologies such as clustered regularly interspaced short palindromic repeats (CRISPR)/Cas9 is being used to accelerate genetic engineering of microbes as it permits rapid and efficient editing of the genome by replacing 20-nucleotide sequences of a chimeric single-guide RNA (sgRNA) complementary to the target sequence of interest [215]. Immediately, the Cas9-sgRNA complex binds to the target DNA sequence, the endonuclease activity of the CRISPR-associated protein (Cas9 protein) cleaves the DNA [215]. This hastens metabolic engineering of proteins and editing of useful genes that could enhance tolerance to inhibitors, or promote utilization of different substrates used for biofuel production. The mechanism and major components of the *Streptococcus pyogenes* Type II CRISPR-Cas9 system have been well characterized. It consists of a Cas9 protein with endonuclease activity which is guided by two types of small RNAs, a target-recognizing CRISPR RNA (crRNA) which binds the target DNA and guides cleavage, and auxiliary non-coding trans-activating crRNAs (tracrRNAs) which base-pairs with the crRNA and permits the formation of Cas9-crRNA complex [216–219]. Genes relevant to biofuel-producing bacterial strains could be edited for better performances, novel genes inserted, or unwanted genes deleted or knocked out. For the activation of specific genes, CRISPR activation (CRISPRa) system is used. In this case, the dCas9 is fused to transcription activators such as RNA Polymerase ɯ subunit present in bacterium such as *Escherichia coli.* To knockout unwanted genes another approach known as CRISPR interference (CRISPRi) which has an inactivated endonuclease activity, is utilized. A catalytically dead Cas9 (dCas9) forms a complex with sgRNA which inhibits RNA polymerase resulting in the blockage of transcription. This arises due to the binding of the dCas9–sgRNA complex to the upstream region of the target gene sequence. Besides, the nuclease-deactivated Cas9 possessing only DNA-binding function guided by sgRNA, has revealed the potential to control regulatory functions in gene expression [220–222]. The CRISPR–Cas9 technology is naturally used by prokaryotes as a defensive mechanism against foreign nucleic acids from viruses or any foreign DNA. Thus, using the CRISPRa and CRISPRi gene-editing technologies, the expression of endogenous genes can be either up-regulated or down-regulated, making it easier for researchers to effectively study the function of genes relevant to metabolic pathways required for biofuel production.

CRISPR-Cas 9 is revolutionizing the science of genetic engineering, and metabolic engineering. Its utilization in genome editing has surpassed that of previous tools such as zinc finger nucleases (ZFNs) and transcription-activator-like effector nucleases (TALENs) previously applied for the genetic manipulation of bacteria [223, 224]. It is now widely used as a genome-editing tool since it is based on RNA–DNA recognition using highly specific 20-nucleotides guide RNA for directing the Cas9 towards the specific site [201]. The versatility of the CRISPR/Cas9 technology is shown by the ease with which it is engineered to enhance the simultaneous targeting of multiple genes for developing potent strains [224, 225]. Several biofuels and other commercial products have been produced by the use of CRISPR-based methods [201, 226]. The CRISPR/Cas9 systems have been employed in the manipulation of genes in several bacterial cells belonging to the genera *Bacillus, Clostridium, Corynebacterium, Escherichia coli, Lactobacillus,Mycobacterium, Pseudomonas, Staphylococcus,* and *Streptomyces *[215]. With this technology, several genetically modified microorganisms with high biofuel-producing abilities have been obtained.

The CRISPR/Cas 9 system was recently used to engineer a dual-operon-based synthetic pathway in the genome of *Escherichia coli* strain MG1655, which produced 5.4 g/L n-butanol in a medium containing glucose as the carbon source and subsequently repeated in an ethanologenic strain of *E. coli* strain SSK42 to produce butanol from xylose using a redox-balanced pathway by Abdelaal et al. [227]. A synthetic butanol cassette was integrated into the genome of *E. coli* strain SSK42 through CRISPR/Cas9 system after removal of the gene encoding endogenous ethanol production. The newly engineered strain ASA02, generated 4.32 g/L butanol in fed-batch fermentation process [227]. *Clostridium saccharoperbutylacetonicum* N1-4 a recognized hyperbutanol-producing strain was edited by targeting two important genes: *pta* and *buk,* which encodes for acetate and butyrate production [228]. Increased butanol production, higher yield, and selectivity of mutants in the batch fermentation were obtained, but this was dependent on the fermentation medium used. The highest butanol yield in the batch fermentation process was obtained in the P2 medium with a yield of 19.0 g/liter [228]. The efficiency was improved using the *PJ23119* promoter to guide RNA (gRNA) expression [228]. In another study, Wasels et al. [229] designed a dual plasmid-inducible CRISPR/Cas9 genome editing tool for the solventogenic strain *Clostridium acetobutylicum* ATCC 824, which led to mutants that produced an isopropanol–butanol–ethanol mixture. Despite the benefits derived from the CRIPSER/Cas9 system in recent times, it requires much expertise and is still in its nascent stage in metabolic engineering, especially in the developing countries.

Advances in high-throughput technologies such as proteomics, transcriptomics, and metabolomics are increasingly been used to understand how specific genes are expressed and the role they play in metabolic pathways associated with biofuel production by lignocellulose degrading microorganisms [230, 231]. Computational tools are often used to obtain a mechanistic understanding of the information derived from these advanced technologies. The use of principal component analysis proteomics-guided engineering led to an improvement in the production of two terpenes by more than 40% via the heterologous mevalonate pathway in *E. coli* [231]. These computational tools and advanced technologies should be fully harnessed for the screening and metabolic engineering of microbial strains for improved industrial production of biofuel.

## Conclusion

Microorganisms are major players in the production of biofuel. However, the product’s yield by native strains is not economical, thus making it necessary to develop and improve them through the approach of metabolic engineering and genetic engineering. Recent studies have focused on applying metabolic engineering to model strain development to optimize high productivity and energy value at a cheaper cost of production. In the nearest future, there is a high possibility that more unique metabolic pathways for biofuel production could emerge from database mining. Thus, the implementation of these pathways in industrial fermentation hosts may overcome any bottlenecks associated with the use of lignocellulosic biomass as a renewable fermentation feedstock. Metabolic engineers need to tap into the use of advanced technologies currently available such as the omic technologies and CRISPER/Cas9 system to design and generate novel strains of microbes with enhanced ability to produce biofuel from diverse substrates by insertion of relevant genes into the genome or deletion of obstructive ones.

## Data Availability

Not applicable.
